# Assessing a Mass-Based Method for the Preparation of Low-Dosed Paediatric Capsules with Baclofen and Spironolactone

**DOI:** 10.3390/pharmacy9010056

**Published:** 2021-03-08

**Authors:** Janosch Klinger, Rolf Daniels

**Affiliations:** Department of Pharmaceutical Technology, Eberhard Karls University, Auf der Morgenstelle 8, 72076 Tuebingen, Germany; janosch.klinger@uni-tuebingen.de

**Keywords:** compounding, pediatrics, capsules, content uniformity, HPLC-UV-Vis

## Abstract

Despite the steadily improving medical care situation in pediatrics, some drugs are still not available in a suitable dose or dosage form and thus need to be prepared extemporaneously. Capsules can be easily compounded at the hospital and public pharmacies, offering an alternative to liquid formulations. This study aims at testing a mass-based approach for the extemporaneous preparation of low-dose pediatric capsules and investigating systematically the API loss during this procedure. A total of 54 capsule batches were prepared with baclofen and spironolactone as pediatric-relevant drugs. The hard capsules were prepared using three different bulking agents consisting of either mannitol, lactose-monohydrate and microcrystalline cellulose mixed with 0.5% colloidal silica. Capsules were tested according to Ph. Eur. method “2.9.40 Content Uniformity” as well as for occurring powder loss and mass uniformity. The results reveal that the mass-based approach, in general, allows the preparation of low-dose pediatric capsules of appropriate quality. However, absolute quality is highly dependent on the homogeneity of the powder mixture and the use of defined parameters for capsule preparation.

## 1. Introduction

In 1968, Shirkey labelled children as therapeutic orphans [1]. For the last few decades, this term has still been accurate, mostly owing to a lack of paediatric drugs and limited research conducted on this topic [2,3,4]. The endeavours of the European Union to overcome this problem and the long-time occurring lack of medicines for children resulted in the Paediatric Regulation, enacted in 2006 [5]. Inspired by the measures undertaken with the Best Pharmaceuticals for Children Act (BPCA) and Pediatric Research Equity Act (PREA) in the United States, the legislative approach obliges pharmaceutical companies to involve children in the earlier stages of medicine development by the means of a Paediatric Investigation Plan (PIP) [6].

Ever since the introduction of this regulation, numerous measures were taken to improve the overall situation of medicines for children. The development of new dosage forms such as orodispersible formulations or multiparticulate systems (for example, mini-tablets and “sprinkles”) led to a paradigm shift from, formerly in paediatrics primarily used, liquid formulations to solid oral dosage forms [7]. Furthermore, the report for the 10th anniversary of the Paediatric Regulation revealed that, from 2007 to 2016, more than 260 new paediatrics had been approved by the European Medicines Agency, underlining the success of the taken measures. Despite those pleasant achievements, the Paediatric Use Marketing Authorization (PUMA) concept, which is also included in the same regulation, sadly failed to meet its expectations [8]. Its aim was to promote the development of medicines, especially for children, for compounds that are off-patent along with reducing frequently practiced off-label use [8,9,10]. Until 2019, only six PUMAs had been granted by the European Medicines Agency (EMA) [11].

A lack of paediatric-licensed formulations leaves a void, which is often filled with medications compounded by pharmacies. The preference on compounded formulations differs throughout Europe. In England and Sweden, liquid formulations are commonly used, whereas in Finland, Italy, and Scotland, powders are used, and in countries like France and Switzerland, capsules are most frequent. In Germany, Spain and Slovenia, a mix of all mentioned formulations is used [12,13]. Compounding is still a controversial issue as it is fraught with risks such as erroneous dosing [14,15]. Neumann and Bureau (2017) tested 56 batches of capsules, containing 1125 units in total, of which only 87.5% passed the acceptance criteria based on the European Pharmacopeia [16].

As stated in a reflection paper published by the EMA, by the age of 6 years most children are able to swallow solid dosage forms [17]. Mini-tablets and “sprinkles” have been proven as suitable dosage forms at younger ages [18,19]. To overcome the problem of swallowability capsules can be opened in order to mix the contained powder with foodstuff [20]. In that case, the capsule itself does not represent the final dosage form but serves as primary packaging for the contained powder.

When it comes to compounding, there is little harmonisation throughout the European Union [21]. Many hospitals have their own standards for the extemporaneous preparation of dosage forms [12]. This lack of standardised methods has a negative impact on the safety and efficacy of extemporaneously prepared formulations [22]. For the last few decades, the preparation method of choice for compounding capsules published by DAC/NRF as well as FNA has mainly been a volume-based procedure. This method is prone to powder and consequently, API loss [23], which is especially critical when preparing low-dose drugs and the API is preferably adsorbed to the instrument’s surfaces.

To overcome this issue, a mass-based method has been elaborated by, amongst others, the DAC/NRF. It is based on the use of a certain bulking agent consisting of mannitol mixed with 0.5% (m/m) with a specified bulk density of 0.50–0.55 g/mL. Furthermore, standardized procedures exist only for few drugs and the applicability is limited to capsule size #1 and micronized APIs. In addition, a 10% overage of the drug is suggested to compensate for the API loss.

The aim of this study was to verify the applicability of the mass-based method and distil information regarding the influence of the filling agent properties. Baclofen and spironolactone were chosen as paediatric-relevant APIs. Baclofen is used in paediatrics for the treatment of spasticity of cerebral palsy [24]. Spironolactone is applicable with congenital heart failure, ascites and nephrotic syndrome [25].

## 2. Materials and Methods

### 2.1. Bulking Agents

Bulking agents were prepared by mixing either microcrystalline cellulose (mcc; PH102), lactose-monohydrate (la) or mannitol (m35; Mannitol 35) (all obtained from Caesar and Loretz GmbH, Hilden, Germany) with 0.5% (m/m) of colloidal silica (Evonik AG, Essen, Germany) manually for 60 s whilst abrading with a powder spreader for three times. Bulk densities $\rho_{b}$ were determined by filling 9–11 g of bulking agent ($m_{f}$) into a graduated 25 mL measuring cylinder without vibration and then reading its volume $V_{f}$. The bulk density was calculated according to Equation (1).

(1) $$\rho_{b}=\;\frac{m_{f}}{V_{f}}$$

For the determination of the compressed density $\rho_{c}\;$, a brass cylinder (weight 267.73 g, length 147 mm, diameter 16.5 mm) was placed on the powder bed five times to level it. The brass cylinder was then placed at the mark 1.5 mL above the levelled powder bed and dropped ten times to compress the powder. The resulting compressed volume $V_{c}$ was used to calculate $\rho_{c}$ according to Equation (2).

(2) $$\rho_{c}=\;\frac{m_{f}}{V_{c}}$$

The density increase due to compression was calculated as follows:

(3) $$\mathrm{density}\;\mathrm{increase}\;\left(\%\right)=\;\frac{\left(\rho_{c}-\rho_{b}\right)}{\rho_{c}}\cdot 100\%$$

Using the values for bulk and compressed density, the amount of bulking agent $m_{b}\;$, that is required for the capsule preparation, was calculated according to Equation (4), which has been empirically derived from preliminary experiments. Hard gelatin capsule shells (Capsugel, Colmar, France) of size #1 were 0.5 mL in volume $V_{C^{\prime }}$ and $n_{C^{\prime }}$ resembles the number of capsules to be prepared.

(4) $$m_{b}=\frac{3\;\cdot \;\rho_{B}+\;\rho_{c}}{4}\;\cdot V_{C^{\prime }}\;\cdot \;n_{C^{\prime }}$$

### 2.2. Capsule Preparation

Capsule batches (n_c_ = 30) of spironolactone (Caesar and Loretz GmbH, Hilden, Germany) containing 0.5 mg, 2 mg and 4 mg and baclofen (Fagron GmbH and Co. KG, Barsbüttel, Germany and Pharmaserv AG, Stansstad, Switzerland) containing 1 mg, 2.5 mg and 4 mg were prepared with an API overage of 10%. The powder mixture was prepared by spreading 1 g of bulking agent in a stainless-steel bowl (diameter 11 cm, volume 300 mL) and placing the API powder on the powder bed. The API was covered with an additional 0.5 g of bulking agent and then mixed for 40 s whilst abrading with a powder spreader twice. One-third of the remaining bulking agent was added and then mixed for 60 s whilst abrading three times with a powder spreader. This step was repeated for the other two-thirds of the bulking agent. The obtained blend was used to fill in clear hard gelatine capsules (Capsugel, Colmar, France) of size #1. The encapsulation process was carried out using a manual capsule filling machine for 30 units (aponorm^®^, WEPA Apothekenbedarf GmbH and Co. KG, Hillscheid, Germany). To this end, powder blend was placed on the upper plate of the filling apparatus with a small scoop and filled in the lower capsule halves with a powder spreader. For distributing the remaining powder mixture into the capsules, the capsule filling device was tapped slightly onto the working bench, to compress the already distributed powder. Empty capsules with a mean net weight of 74 mg were numbered to trace back their position on the capsule filling machine.

The compositions of the investigated capsules are shown in Table 1.

### 2.3. Sample Preparation

Each capsule was emptied into a separate 15 mL Falcon^®^ tube and mixed with a solvent mixture consisting of 60% water and 40% acetonitrile (solvent mixture A). Capsule contents were removed by slightly squeezing each capsule half and tapping it repeatedly against the edge of the tube orifice. Capsule halves were checked to be visually clean as an indicator for complete emptying. The tubes were vortexed (Vortex 2, Scientific Industries inc., Bohemia, NY, USA) and subsequently shake-mixed (VXR basic Vibrax^®^, IKA-Werke, Staufen im Breisgau, Germany) for 60 min at 1000 rpm followed by centrifugation (Megafuge 1.0R, Heraeus, Hanau, Germany) for 5 min at 3000 rpm. Samples were filtered through a 0.2 µm PTFE-filter (Macherey-Nagel GmbH and Co. KG, Düren, Germany) prior to HPLC assay.

### 2.4. Residual API on Working Materials

Residual API on pestle, powder spreader, mixing vessel, capsule filling machine and the spoon, that has been used for transferring the powder mixture, were sampled with cotton swabs wettened with 1 mL of solvent mixture A. If possible, devices were rinsed with the same solvent mixture with a defined volume (see Table 2). The number of used cotton swabs depended on the size of the device to be investigated. Rinsing solution and swabs were collected in separated 50 mL Falcon^®^ tubes and vortexed for 10 s. Samples were then treated in accordance with the method described previously.

### 2.5. HPLC-UV-Vis Assay

All capsules were analysed for their API content by UV-Vis-HPLC using a Shimadzu LC-20A prominence (Shimadzu, Duisburg, Germany) equipped with a UV-Vis detector. Spironolactone assays were carried out referring to Sandall et al. (2006) [26]. A Nucleosil^®^ 100—5 C18 125 × 4 mm, 5 µm (Macherey-Nagel GmbH and Co. KG, Düren, Germany) was used with mobile phase A and B consisting of water and MeOH (Sigma-Aldrich, Steinheim, Germany) respectively. The flow was isocratic with a ratio of 60:40 (*v*/*v*) (A/B) at a flow rate of 0.8 mL min^−1^. The oven temperature was kept at 40 °C and 10 µL were injected. Spironolactone was detected at a wavelength of 254 nm. Referring to a method of Dukova et al. (2015) [27], baclofen was quantified using a Nucleodur^®^ 100-5 C18 Gravity 125 × 4 mm, 5 µm (Macherey-Nagel GmbH and Co. KG, Düren, Germany). Mobile phase A and B consisted of water with a pH adjusted to 9 with triethylamine (VWR International S.A.S, Fontenay-sous-Bois, France) and acetonitrile, respectively. An isocratic flow with a ratio of 90:10 (*v*/*v*) (A/B) was chosen at a flow rate of 1.0 mL/min. Baclofen was detected at 220 nm and the injection volume was 10 µL. All 30 capsules of each batch were analysed.

### 2.6. Quality Parameters

Content uniformity was evaluated according to Ph. Eur. method “2.9.40 Uniformity of Dosage Units”. Specifications were met when the calculated acceptance value was below 15. Calculations were carried out with a *k*-factor of 2.4 for T = 100%. Furthermore, the mass loss that occurred during preparation was determined, which, according to the DAC/NRF, should not exceed a limit of 3%. The percent mass loss was calculated according to Equation (5), where $m_{t}$ is the target mass that includes the sum of the initially weighed fraction of bulking agent and API, $m_{a}$ is the actual weight of the prepared capsules and $m_{c}$ is the average weight of the empty capsule shells.

(5) $$\frac{m_{t}*\left(m_{a}-m_{c}\right)}{m_{t}}*100$$

The deviation of the capsule masses was also considered and should be below 5% as suggested by DAC/NRF.

Mixing homogeneity was assessed by the standard deviation of the quotients of the capsule content and the mass of each capsule.

### 2.7. Particle Size Analysis

Particle size analysis was carried out according to Ph. Eur. method “2.9.31 Particle size analysis by laser diffraction” using a Mastersizer 2000 (Malvern Instruments GmbH, Herrenberg, Germany) equipped with a Scirocco 2000 powder module. The apparatus covers a range of 0.2–2000 µm.

The dispersion pressure was kept at 2.5 bar, obscuration was held between 1–8%. For m35, a feed rate of 56% was chosen whereas la and mcc were examined with a feed rate of 80%.

Results were evaluated according to the Mie theory, choosing a refraction index of 1.52 and an adsorption index of 0.1.

### 2.8. SEM Images

The samples of interest were attached to an aluminium rivet with double-faced adhesive tape and sputtered with gold (0.04 mbar, accelerating voltage: 2.1 kV, current 20 mA, sputter time: 4 × 60s). SEM images were taken at an accelerating voltage of 5 kV with a Zeiss DSM 940 A (Carl Zeiss Microscopy GmbH, Göttingen, Germany) and an Orion 5.25 (Rauscher GmbH, Munich, Germany).

### 2.9. Raman Spectroscopy

Spectra were taken using a confocal Raman microscope (alpha 500R, WiTec GmbH, Ulm, Germany) equipped with a laser (excitation wavelength 523 nm), a UHTS 300 spectrometer (1800 g/mm grid) and a DV401-BV CCD camera. A 40-fold 0.6 NA objective in combination with a 50 µm glass fiber was used. Recorded spectra covered a spectral range from 0–4000 cm^−1^ at a resolution of 4 cm^−1^. Spectra were recorded in an area of 50 × 50 µm with a step size of 0.3 µm. Integration time was 0.05 s.

Reference spectra were taken from the pure substance with an integration time of 0.01 s. Images were generated using Project Plus 4 Software (WiTec GmbH, Ulm, Germany).

## 3. Results

### 3.1. Characterization of Bulking Agents

#### 3.1.1. SEM Images

**Figure 1:**
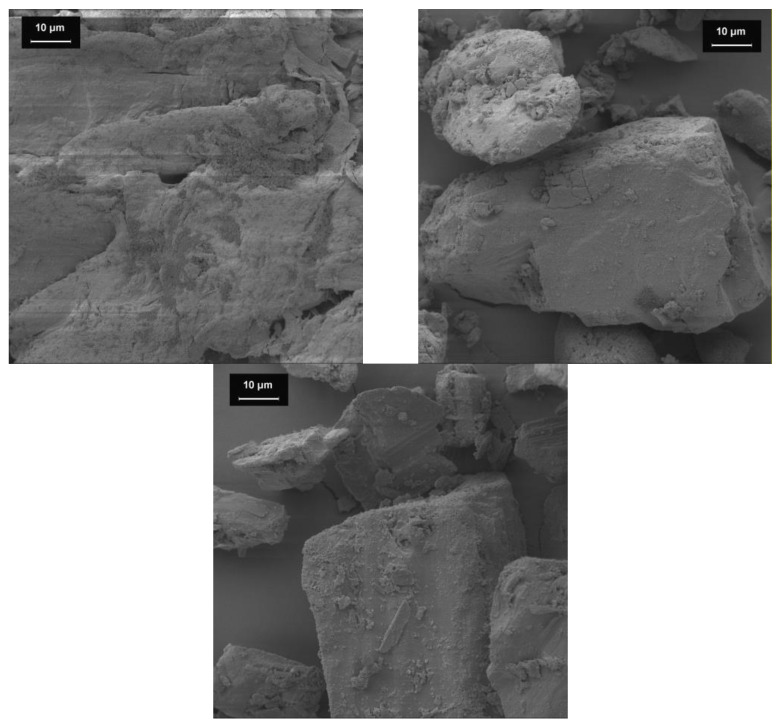
SEM images of the bulking agents (1000-fold magnification). Bulking agents comprising mcc (**top left**), la (**top right**) and m35 (**lower middle**).

SEM images (Figure 1) show all three bulking agents after being processed with highly dispersed silicon dioxide. As expected, agglomerates of silicon dioxide can be spotted on the particle surface. For mcc, an accumulation of anhydrous silica in the pores of the filling agent particles can be observed.

#### 3.1.2. Particle Size Analysis

**Figure 2:**
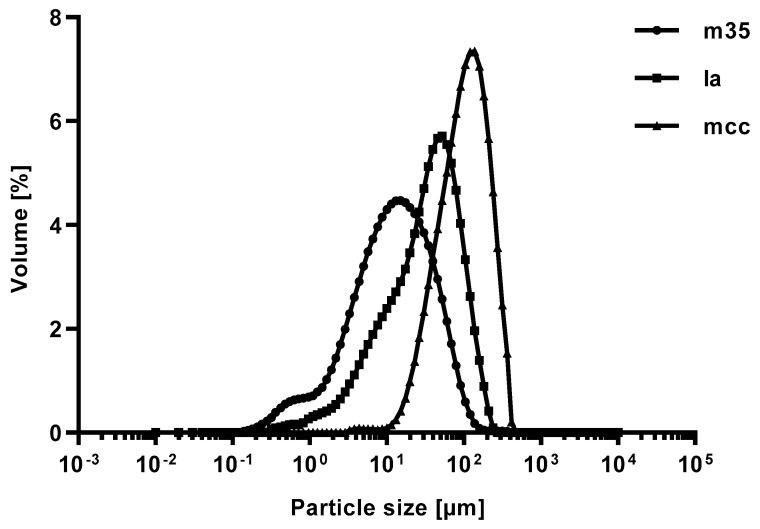
Volume fraction of the particle size distribution of bulking agents comprising mcc (**top left**), la (**top right**) and m35 (**lower middle**). Measured by laser diffraction.

The results reflect the diversity of the individual bulking agents. The bulking agent comprising m35 has a specified nominal particle size of 50 µm (determined by laser diffraction) [28], although a considerable portion of the powder consists of very fine particles as can be seen on the corresponding knee of the curve (Figure 2). The fine-grained proportion below 1 µm accounts for a volume fraction of approximately 0.7% which is also confirmed by the Dv(0.1)—value of 2.12 µm.

The bulking agent consisting of la shows a broader, shifted particle size distribution with bigger particles compared to m35 (Dv(0.9) = 116.94 µm).

The used grade of mcc represents the largest particle size and a lower fraction of fine particles as opposed to m35 and la, as elucidated by the Dv(0.9) of 255.96 µm and the Dv(0.1) of 38.27 µm, respectively. The shoulder of the corresponding density distribution function between 1 and 10 µm displays a portion of fine particles. A monomodal particle size distribution could not be observed. Average particle sizes by volume at the undersize values of 90, 50, and 10 per cent (Dv(0.9), Dv(0.5), Dv(0.1)) of the used bulking agents are summarized in Table 3.

#### 3.1.3. Determination of the Compressed Density and the Amount of Bulking Agent

The differences between the mean values of the bulk and compressed density of the used filling agents are shown in Table 4. Bulking agents comprising m35 and la revealed a high bulk density of 0.531 g/mL and 0.564 g/mL, respectively, that could be compressed by approximately one-fourth. The bulking agent prepared with mcc has a bulk density of 0.392 g/mL which is the lowest bulk density of all fillers which could only slightly be increased by 11.3 ± 1.64% through the compression procedure. Consequently, the calculated mass of powder that is necessary to fill each capsule (Equation (4)) is low compared to m35 and la.

### 3.2. Characterization of Spironolactone and Baclofen

#### 3.2.1. Baclofen

**Figure 3:**
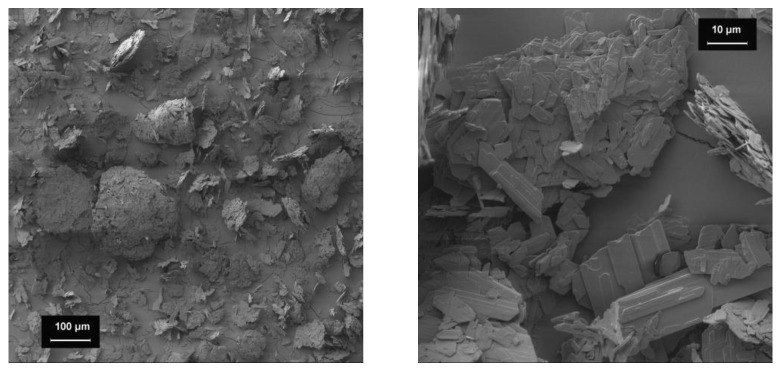
SEM-images of baclofen;100-fold magnification (**left**), 1000-fold magnification (**right**).

Figure 3 depicts eminently flat particles with a smooth surface, to which smaller fragments of baclofen adhere. According to the SEM-images, a broad particle size distribution can be assumed with only a few particles being smaller than 100 µm.

#### 3.2.2. Spironolactone

**Figure 4:**
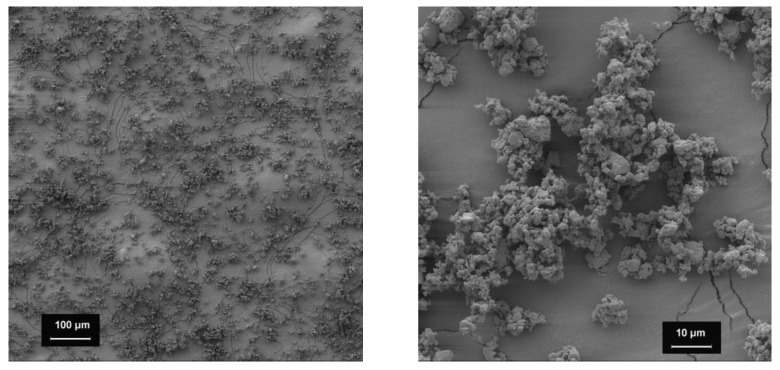
SEM-images of spironolactone; 100-fold magnification (**left**), 1000-fold magnification (**right**).

The spironolactone powder used in this study consisted of small primary particles with a size below 10 µm but a high tendency to agglomerate (Figure 4). Surface roughness is enhanced by the adhesion of smaller particle fragments. SEM-images depict a uniform particle size distribution. In combination with coarser filling agent particles, the formation of interactive mixtures is possible.

### 3.3. Raman Images

#### 3.3.1. Baclofen

**Figure 5:**
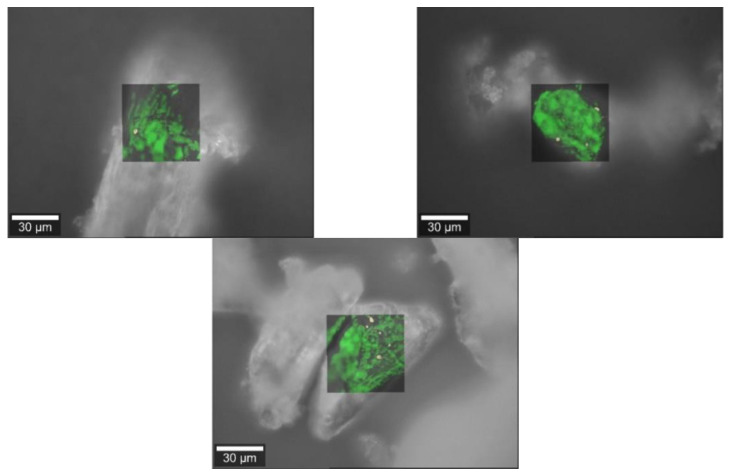
Raman images of baclofen (yellow) and the bulking agents (green) comprising m35 (**top left**), la (**top right**) and mcc (**bottom middle**).

Figure 5 clearly shows that there is only a small proportion of API adhering to the surface of the filling agent particles. This is owed to the particle size of baclofen which, with a Dv(0.9) of 40 µm, could not be considered a micronized API. Therefore, the formation of an interactive mixture with the used powder blends was unlikely.

#### 3.3.2. Spironolactone

**Figure 6:**
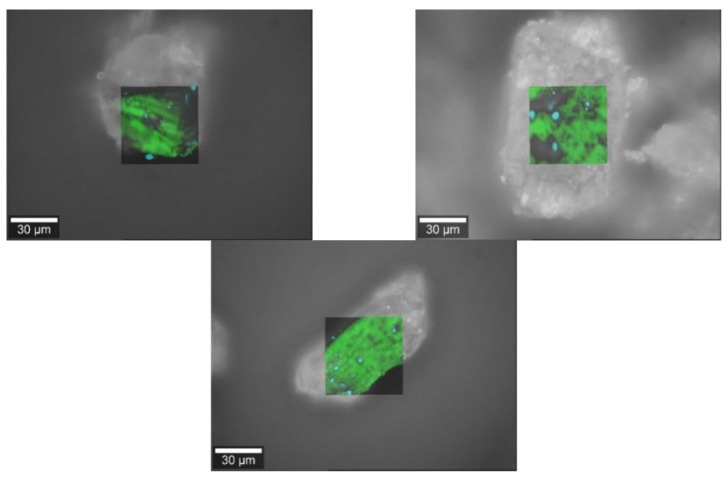
Raman images of spironolactone (cyan) and the bulking agents (green) comprising m35 (**top left**), la (**top right**) and mcc (**bottom middle**).

Raman images (Figure 6) reveal the adhesion of spironolactone to the surface of the filling agents m35, la and mcc. The formerly agglomerated API particles could be segregated through the mixing process generating an interactive mixture with the bulking agents.

### 3.4. Capsule Quality

#### 3.4.1. Baclofen Capsules

##### Baclofen 1 mg

**Figure 7:**
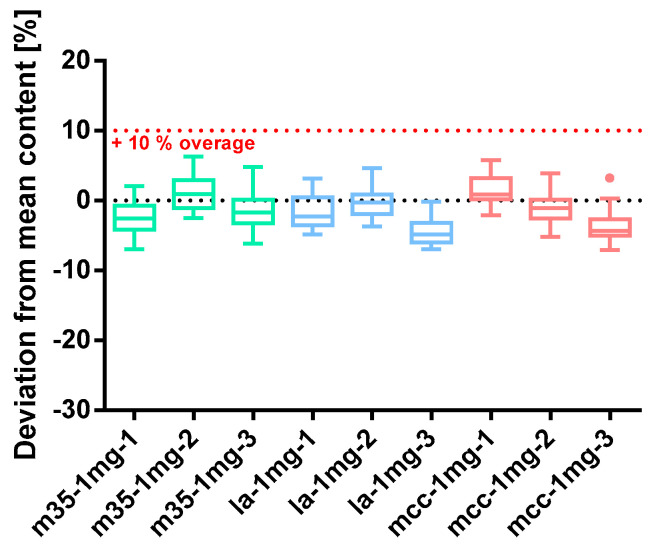
Deviation from the mean content of baclofen 1 mg capsule batches prepared with bulking agents comprising m35, la and mcc; Tukey’s box plot: □ box—includes 50% of all data points and shows the median (line in the box), the length of the box resembles the interquartile range (IQR); T whisker—includes values within the 1.5-fold IQR; ● outlier; *n* = 30.

Figure 7 shows the deviation from the mean capsule content of the baclofen 1 mg capsule batches prepared with 10% overage. Regardless of the used bulking agent, the contents of all capsules in each batch are distributed narrowly around the targeted content of 1 mg. The whiskers of the box plot graph elucidate that there are no obvious outliers. However, seven of nine batches have a median content below the targeted 1 mg, albeit it is marginally.

The other quality parameters are listed in Table 4. All capsules pass the test of content uniformity (2.9.40 Ph. Eur.) as the calculated acceptance value AV is less than 15. Capsules prepared with mcc filling agent exceed the suggested threshold of a powder loss of 3%. When preparing batch mcc*-1mg-3* the, with 7.27%, the highest mass loss occurred. The filling of the capsules happened according to the 5% deviation limit. The maximum deviation of 2.02% was obtained when preparing batch m35*-1mg-1* with m35 as a filling agent. The mean capsule content and its deviation are shown in Table 5, underlining the quality of the resulting baclofen 1 mg capsules. Powder homogeneity can be regarded as given for all prepared batches due to the low values of SD_rel_ content/mass.

##### Baclofen 2.5 mg

**Figure 8:**
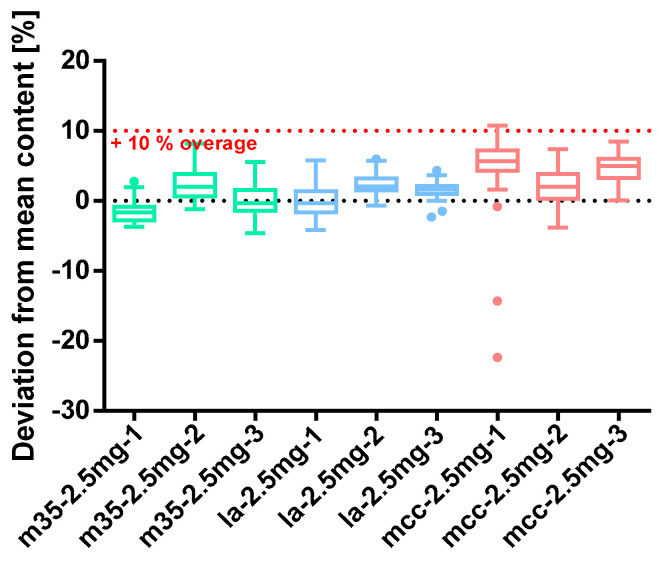
Deviation from the mean content of baclofen 2.5 mg capsule batches prepared with bulking agents comprising m35, la and mcc; Tukey’s box plot: □ box—includes 50% of all data points and shows the median (line in the box), the length of the box resembles the interquartile range (IQR); T whisker—includes values within the 1.5-fold IQR; ● outlier; *n* = 30.

Baclofen 2.5 mg capsules were close to the target content when m35 and la as filling agents were used, and a 10% overage was applied. All capsules with mcc as bulking agent yielded a median and mean content that exceeded the target values of 2.5 mg. However, even in these cases, parts of the 10% overage were lost. Batch mcc*-2.5mg-1* comprised two severely underdosed capsules (−22.34 and −14.29%) marked as outliers in Figure 8. Owed to these circumstances this batch does not comply with the test of uniformity of dosage units (Ph. Eur. 2.9.40.) as its AV is at 16.07 (see Table 6). An inhomogeneous powder mixture is the most probable reason for this. All other batches revealed an AV below 15 and therefore met the Ph. Eur. requirements. Three batches slightly exceed the targeted maximum powder loss of 3% with a maximum of 4.67% for batch mcc*-2.5mg-2*. Surprisingly, the other capsule quality parameters were not affected. All capsules were filled uniformly as the standard deviation of the mass is <5%. The API was likewise evenly dispensed between the capsules except for batch mcc*-2.5mg-1* that also shows the highest SD_rel_ of the content-mass-ratio.

##### Baclofen 4 mg

**Figure 9:**
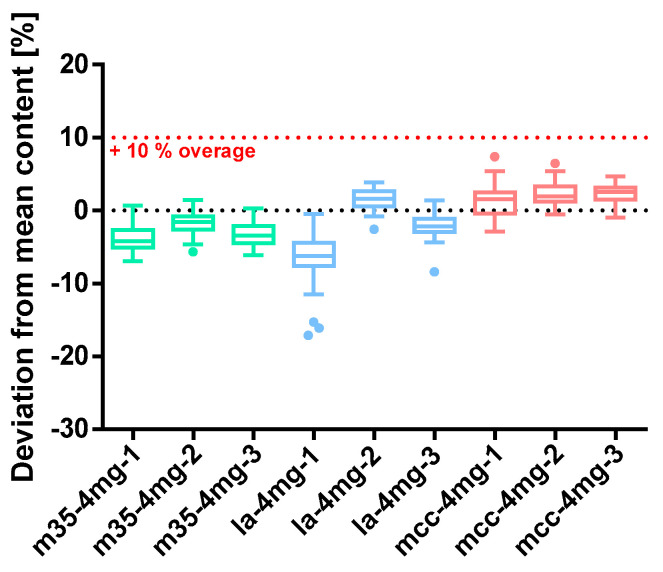
Deviation from the mean content of baclofen 4 mg capsule batches prepared with bulking agents comprising m35, la and mcc; Tukey’s box plot: □ box—includes 50% of all data points and shows the median (line in the box), the length of the box resembles the interquartile range (IQR); T whisker—includes values within the 1.5-fold IQR; ● outlier; *n* = 30.

The capsules prepared with 4 mg of baclofen showed consistent results for all m35-batches. As can be seen in the box plot chart in Figure 9, their median content was constantly below the target content justifying the applied 10% overage. This was opposite to the findings for capsules prepared with la as a filling agent. Batch la*-4mg-1* seems to be highly underdosed and the individual values are scattered in a wide range. Furthermore, three outliers were identified and depicted as dots between −15.28 and −17.11%. The second and the third la-batch had a median and mean content close to the target value of 4 mg, although one capsule of *la-4mg-3* lacks API as indicated by the outlier at −8.38% in the corresponding boxplot chart. Batches prepared with mcc as a filling agent show consistent results albeit all median and means content exceeds 4 mg slightly.

All baclofen 4 mg capsule batches complied with the test of uniformity of dosage units (Ph. Eur. 2.9.40.). Interestingly, the powder loss exceeded the limit of 3% in all cases. Batch *la-4mg-1* revealed an AV of 13.16 and with 3.73 mg the lowest overall capsule content while the mass loss only amounts to 3.16%. Capsules were filled uniformly with all mass deviations below 3% which can be obtained from Table 7. The deviation of the capsule content was adequate except for batch la*-1mg-1* where the distribution was wide and three outliers occurred, explaining the unsatisfying outcome of the AV. Taking into account the SD_rel_ of the ratio of capsule content over capsule mass, this was most likely due to a slight inhomogeneity of the powder mixture.

#### 3.4.2. Spironolactone Capsules

##### Spironolactone 0.5 mg

**Figure 10:**
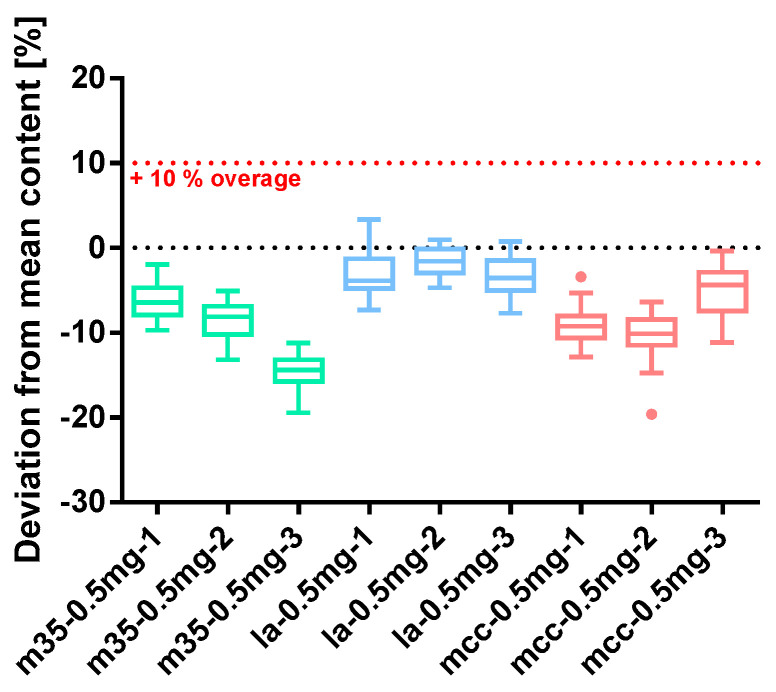
Deviation from the mean content of spironolactone 0.5 mg capsule batches prepared with bulking agents comprising m35, la and mcc; Tukey’s box plot: □ box—includes 50°% of all data points and shows the median (line in the box), the length of the box resembles the interquartile range (IQR); T whisker—includes values within the 1.5-fold IQR; ● outlier; *n* = 30.

Capsules prepared with 0.5 mg of spironolactone showed inconsistent results (Figure 10). All three batches prepared with m35-filling agent were largely underdosed although prepared with a 10% overage. This is owed to a high powder loss, which was most pronounced with the batches m35*-0.5mg-2* and m35*-0.5mg-3*, where the powder loss amounts to 10.67% and 10.84%, respectively. Spironolactone 0.5 mg capsules prepared with mcc based bulking agent were underdosed as well, however, without observing a distinctive powder loss. Batch mcc*-0.5mg-2* did not exceed the suggested 3% threshold for powder loss and still showed underdosed capsules with an outlier that deviates by −19.62%. Capsules prepared with the la bulking agent are slightly underdosed and show a comparably low powder loss that still exceeded the 3% limit in two cases (see Table 8). However, despite deficiencies regarding their overall content, all batches except m35*-0.5mg-3* met the requirements for the Ph. Eur. content uniformity test indicating that the capsules were filled uniformly, and their content was consistent. Powder homogeneity could additionally be proven for all batches of which mcc*-0.5mg-2* with a SD_rel_ content/mass of 3.05% showed the least homogenous powder blend.

##### Spironolactone 2 mg

**Figure 11:**
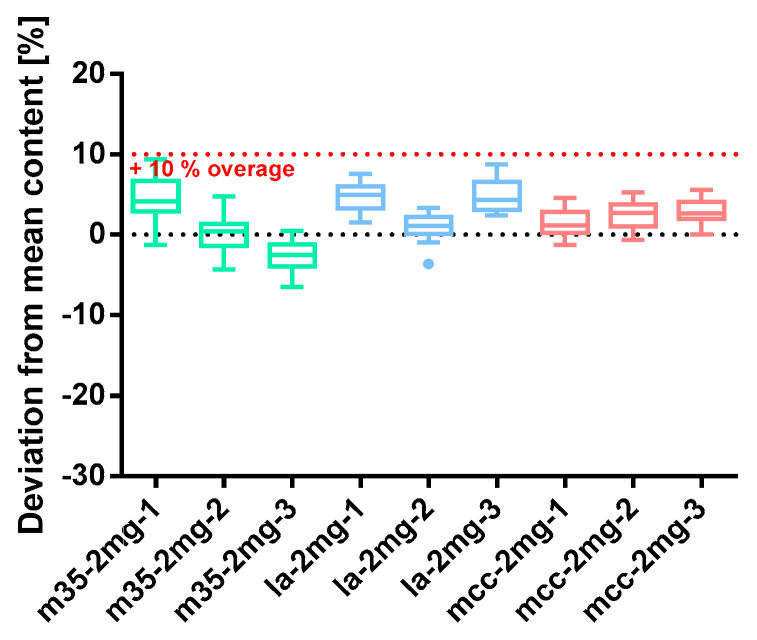
Deviation from the mean content of spironolactone 2 mg capsule batches prepared with bulking agents comprising m35, la and mcc; Tukey’s box plot: □ box—includes 50°% of all data points and shows the median (line in the box), the length of the box resembles the interquartile range (IQR); T whisker—includes values within the 1.5-fold IQR; ● outlier; *n* = 30.

Spironolactone 2 mg capsule batches were comparably prepared by using the three different kinds of bulking agents and a 10% overage. Batches with m35 bulking agents revealed highly inconsistent results (Figure 11). Spironolactone content and powder loss were not related. While the median content per capsule of m35*-2mg-1* was above the target content at a powder loss of 3.16%, the powder loss of m35*-2mg-2* was at 4.66% nonetheless meeting the target content. Furthermore, batch m35*-2mg-3* was slightly underdosed with a powder loss (4.25%) in between the others (see Table 9). Two of three batches prepared with la as a filling agent were overdoses while the powder loss of all three batches did not exceed the 3% limit. The capsules prepared with the bulking agent comprising mcc showed similar results. All of them were marginally overdosed and except mcc*-2mg-3* with a powder loss <3%. All spironolactone 2 mg capsule batches complied with the requirements for content uniformity specified in Ph. Eur. capsules were filled uniformly, and the mass deviation did not exceed 5%. Furthermore, powder homogeneity could be proven for all batches.

##### Spironolactone 4 mg

**Figure 12:**
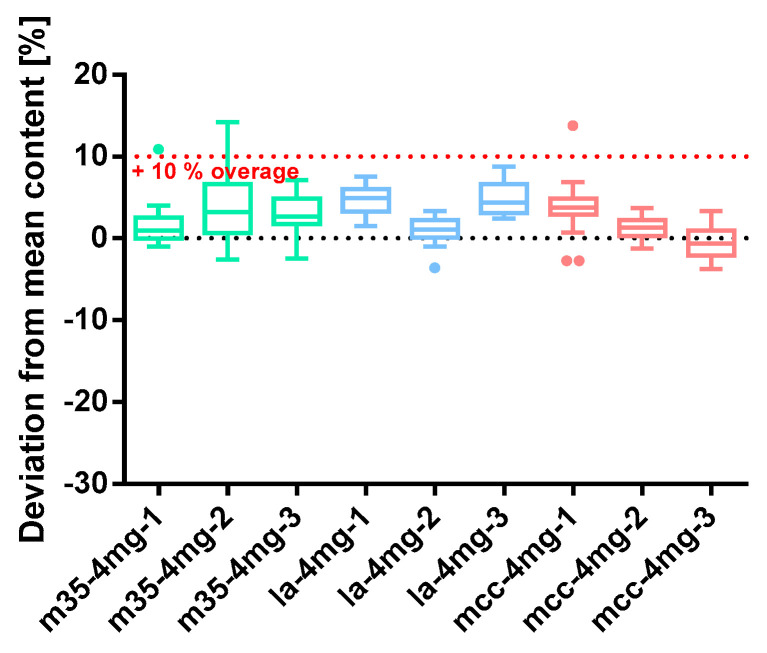
Deviation from the mean content spironolactone 4 mg capsule batches prepared with bulking agents comprising m35, la and mcc; Tukey’s box plot: □ box—includes 50°% of all data points and shows the median (line in the box), the length of the box resembles the interquartile range (IQR); T whisker—includes values within the 1.5-fold IQR; ● outlier; *n* = 30.

The results for the spironolactone 4 mg capsules are shown in Figure 12 and Table 10. Figure 12 depicts an extremely wide distribution of the individual capsule contents for m35*-4mg-2* that came along with a high deviation of 4.06% in the capsule mass. The powder loss was lower than the 3% limit, explaining the fact that these capsules were overdoses. Altogether, this led to an AV of 10.59 that, surprisingly, still met the requirements of the content uniformity test. Batches m35*-4mg-1* and m35*-4mg-2* are as well slightly overdosed, but the individual values did not scatter very much. The first batch of capsules prepared with the bulking agent comprising la showed a low powder loss of 2.20% yielding capsules with a mean spironolactone content of 4.34 mg. Although the capsule masses varied in a similar range as the capsule content, a high AV of 13.61 was obtained. Batches la*-4mg-2* and la*-4mg-3* complied with the criteria for powder loss and mass homogeneity. However, batch la*-4mg-2* showed a content right at the target value whereas batch la*-4mg-3* was an overdose, marginally. From the batches with mcc-filling agent, mcc*-4mg-1* exhibited three statistical outliers that are owed to the very low overall deviation of the capsule content. The powder loss of all three batches exceeded the 3% threshold. Nevertheless, this did not notably affect the capsule’s quality attributes. Capsules are filled uniformly with a deviation of the mass <1%. All prepared spironolactone 4 mg capsule batches passed the content uniformity test (Ph. Eur. 2.9.40). The homogeneity of the powder blend can be assumed as given according to SD_rel_ content/mass (Figure 12).

### 3.5. Residual API on Equipment

#### 3.5.1. Baclofen Capsules

**Figure 13:**
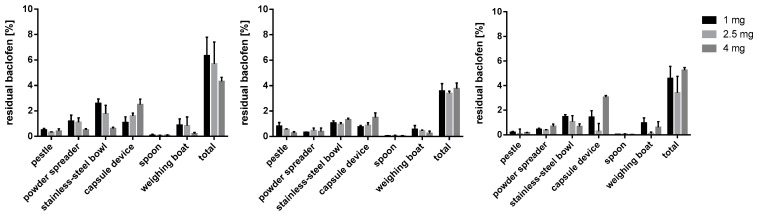
Baclofen residues on used equipment; m35 (**left**), la (**middle**), mcc (**right**); *n* = 3.

The complete equipment used was tested for adhering baclofen after capsule preparation. Figure 13 exhibits the percentage of adhering baclofen in relation to initially weighed out API. When m35 was the chosen filling agent the API relative loss decreased with higher dosing from 6.43 ± 1.44% at 1 mg to 4.32 ± 0.31% at 4 mg. Most baclofen adhered to the stainless-steel mixing bowl and the capsule filling device depending on the target dose. Furthermore, for m35 a comparably high fraction of baclofen adhered to the powder spreader at lower doses (1 mg and 2 mg; see Figure 13). Comparing the residues on the stainless-steel bowl and the capsule filling device, the residues for each dose were in adverse order. While residues are decreasing with an increasing dose for the mixing vessel, a higher percentage of baclofen adhered to the capsule filling device. Pestle, spoon, and the weighing boat did not significantly add to the overall residues. It is worth to note, that the residues on the weighing boat were solely dependent on the API itself and were investigated for the sake of completeness.

For the bulking agent comprising la, the lowest overall loss of baclofen could be observed. Stainless-steel bowl and capsule device showed a higher tendency for adhering API, with maximum values for 4 mg capsules at 1.77 ± 0.66% and 1.62 ± 0.21%, respectively. The percentage amount of lost baclofen was not clearly affected by an increasing capsule dose.

When using mcc as a filling agent a decreasing percentage of API adhered to the mixing bowl with increasing dose (see Figure 13). In accordance with the findings for m35, a reverse trend was found for the capsule filling device. For baclofen 4 mg capsules a loss of 3.07% ± 0.13% could be detected for the capsule device. Low or almost negligible residues of baclofen were recovered from pestle, powder spreader and spoon.

The overall baclofen residues on the equipment ranged from 3.39 to 6.43%. The lowest values were seen with la as filling agent whereas m35 promoted adhesion of baclofen.

#### 3.5.2. Spironolactone Capsules

**Figure 14:**
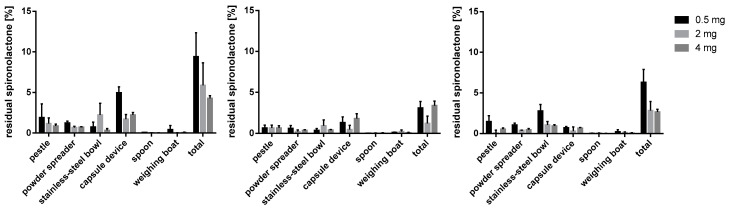
Spironolactone residues on used working materials; m35 (**left**), la (**middle**), mcc (**right**); *n* = 3.

Spironolactone residues (Figure 14) were most pronounced with m35 as filling agent and amount to 9.41 ± 2.95% for batches with 0.5 mg API. Furthermore, the unusually high powder loss played a major role in the spironolactone residues. For the 0.5 mg dose, most of the API was lost on the capsule device along with the pestle (4.96 ± 0.73% and 1.91 ± 1.67%). During the preparation of capsules with 2 mg spironolactone, a high proportion of residues could be registered on the stainless-steel bowl. The remaining materials like powder spreader, spoon and weighing boat showed a marginal impact.

For batches prepared with bulking agent consisting of la, an overall low total loss of API could be shown. The most withheld amount of spironolactone could be detected on the capsule device. Interestingly, the residues were the lowest when preparing capsules with 2 mg dosing.

The residues for the preparation of capsules with mcc as a filling agent showed the highest percentage loss of API for the lowest dose of 0.5 mg (6.30 ± 1.58%). This observation is according to the batches prepared with m35 as a filling agent, although the powder loss for mcc-capsules was less pronounced. The highest individual loss could be detected on the stainless-steel bowl for 0.5 mg capsules with 2.80 ± 0.78%.

### 3.6. Residues on Capsule Shells

**Figure 15:**
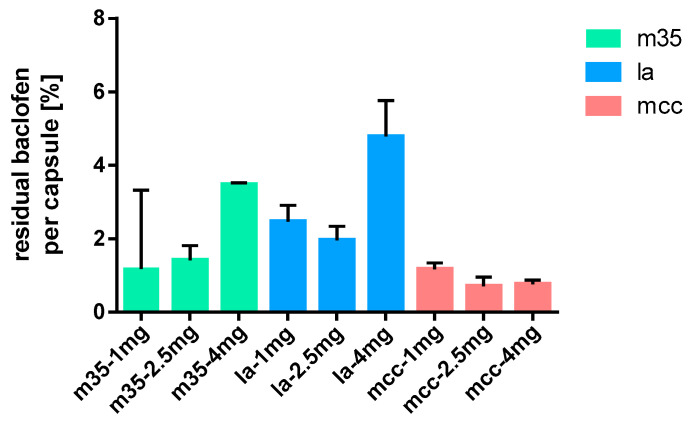
Baclofen residues on capsule shells after emptying; *n* = 15.

**Figure 16:**
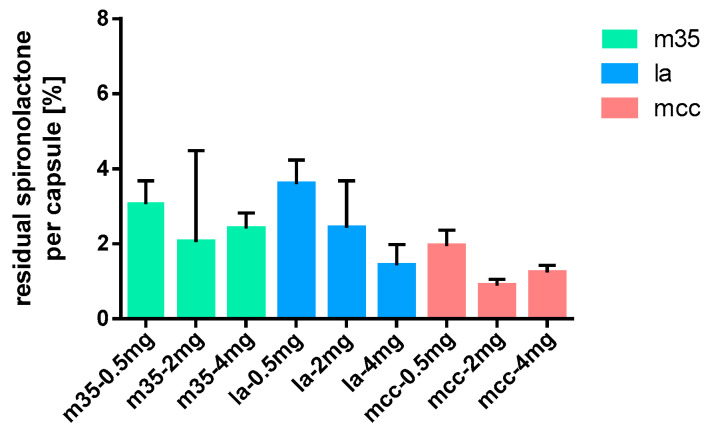
Spironolactone residues on capsule shells after depletion; *n* = 15.

As paediatric capsules are intended to be used by opening and adding the powder to food or drinks, it was of interest how much API adhered to the capsule shell after emptying. Figure 15 and Figure 16 show the percentage of retained API in relation to the respective dose. A higher percentage of baclofen remained in the capsules when m35 or la were used as filling agents compared to mcc. This observation was most pronounced for the baclofen 4 mg capsules. Interestingly, a clear relationship between dose and residual baclofen could not be extracted from the obtained data (Figure 15).

Figure 16 shows that the highest percentage of spironolactone adhered to the capsule shells for the 0.5 mg doses. Moreover, mcc proved to be superior as filling agent to la and m35, where 3.60 ± 0.63% and 3.07 ± 0.62%, respectively, of the target dose were not available. For higher doses, the relative amount of API seemed to decrease, although there was no obvious trend. This is consistent with the observation made for adhesion of baclofen to the capsule shells.

## 4. Discussion

Presently, the mass-based method implemented by the DAC/NRF laboratory represents a kind of standard for the extemporaneous preparation of low-dose paediatric capsules. Besides other studies, the results of this study confirm the applicability of this method. However, the restriction to one singular specified bulking agent might be a shortcoming for general use. This could be resolved by introducing a new formula that allows us to calculate the amount of required bulking agent by the means of the bulk density and a “new” *compressed* density. Both can be easily determined extemporaneously at a public or hospital pharmacy without requiring sophisticated equipment. The applicability was tested for two APIs, namely baclofen and spironolactone, and three bulking agents comprising la, m35 and mcc plus 0.5% colloidal anhydrous silica as a glidant.

For the used grade of spironolactone, the formation of interactive mixtures with all three different bulking agents could be visualized by means of Raman imaging. In contrast, only a small fraction of baclofen adhered to the particle surface of the filling agents due to its substantially larger particle size. However, experimental data did not reveal a better performance for either the interactive or the non-interactive mixture with regards to the defined quality parameters.

In total 54 batches of capsules with baclofen and spironolactone referring to their therapeutic doses for neonates [24,25] were prepared by skilled persons and evaluated according to defined quality parameters. From these 54 batches, 52 complied to the Ph. Eur. content uniformity testing except for two. Baclofen batch mcc*-2.5mg-1* did not pass the test due to an inhomogeneous powder mixture. Powder homogeneity for the remaining batches was consistently acceptable. For spironolactone batch m35*-0.5mg-1*, the occurring powder loss exceeded the tolerance of the method by far and led to severely underdosed capsules. Altogether 22 of 54 batches exceeded the 3% powder loss limit suggested by DAC/NRF without impairing the pharmacopeial quality attributes. Obviously, utilizing a 10% overage of API compensates satisfactorily for mass loss that occurs during the practical routine. Further investigations including a larger number of laboratories may be necessary to validate the so far suggested 3% threshold for powder loss. The deviation of all capsule masses was always below the targeted 5%, thus it is clearly possible to fill the capsules uniformly even though the powder needs to be compressed by tapping manually.

A statistically proven superiority of a certain bulking agent was not found as the coefficient of variation of the individual values was too high. This is owed to the manual preparation which has inherently a limited reproducibility and the overall small differences between the tested bulking agents. However, it could be observed that only when la was used as a building agent all capsules passed the Ph. Eur. content uniformity test.

With respect to the equipment, mixing bowl and capsule filling retained by far the highest fraction for both APIs although for baclofen the effect was more pronounced. This can be easily attributed to their larger surface area compared to the pestle, powder spreader, spoon and weighing boat offering more space for the powder to adhere. Furthermore, the contact with the powder mixture is more intense making it more likely that the API particles adhere. Nonetheless, an API dependency is to be assumed as for hydrocortisone high API portions were found on the pestle and the powder spreader in previous research [29].

The percentage residues on the capsule shells can be assumed as the lowest for bulking agents consisting of mcc. For spironolactone, a higher portion of the target dose seems to adhere when the dose is 0.5 mg compared to 2 mg and 4 mg. One should keep in mind that, when capsules are not being emptied thoroughly, a higher proportion might stay inside the capsule shell, affecting the overall dose.

## 5. Conclusions

In conclusion, the extemporaneous preparation of low-dose paediatric capsules of sufficient quality can be assured. In contrast to the method of DAC/NRF which can only be applied using a certain specified bulking agent, the elaborated method offers a straightforward way to compound low-dosed capsules using powdered API and a variety of bulking agents. The proposed method for characterising the bulking agent is simple and can be performed without extensive equipment. Although this study has only proven the applicability for spironolactone and baclofen and three bulking agents (m35, *la*, mcc), it seems to be very likely that the supposed methodology can be used for most APIs and bulking agents. To prove this, further investigations with other APIs are desirable for making the extemporaneous preparation of capsules as safe as possible for patients and assure the best quality possible.

## Figures and Tables

**Table 1:** Composition of the powder blends used to prepare 30 capsules including a 10% overage of the API.

	API [mg]	m35 [mg]	la [mg]	mcc [mg]	Colloidal Silica [mg]
Baclofen 1 mg Capsules	33.0	8574.9			43.1
Baclofen 1 mg Capsules	33.0		9227.6		46.6
Baclofen 1 mg Capsules	33.0			6040.6	30.4
Baclofen 2.5 mg Capsules	82.5	8574.9			43.1
Baclofen 2.5 mg Capsules	82.5		9227.6		46.6
Baclofen 2.5 mg Capsules	82.5			6040.6	30.4
Baclofen 4 mg Capsules	132.0	8574.9			43.1
Baclofen 4 mg Capsules	132.0		9227.6		46.6
Baclofen 4 mg Capsules	132.0			6040.6	30.4
Spironolactone 0.5 mg Capsules	16.5	8574.9			43.1
Spironolactone 0.5 mg Capsules	16.5		9227.6		46.6
Spironolactone 0.5 mg Capsules	16.5			6040.6	30.4
Spironolactone 1 mg Capsules	33.0	8574.9			43.1
Spironolactone 1 mg Capsules	33.0		9227.6		46.6
Spironolactone 1 mg Capsules	33.0			6040.6	30.4
Spironolactone 4 mg Capsules	132.0	8574.9			43.1
Spironolactone 4 mg Capsules	132.0		9227.6		46.6
Spironolactone 4 mg Capsules	132.0			6040.6	30.4

**Table 2:** On residual API investigated devices with the amount of used extraction volume and cotton swabs.

Device	Extraction Volume [mL]	Number of Used Swabs	Total Volume [mL]
Weighing boat	5	-	5
Spatula	5	-	5
Pestle	10	2	12
Powder spreader	10	2	12
Mixing bowl	15	3	18
Capsule filling machine	19	6	25

**Table 3:** Average particle sizes by volume at the undersize values of 90, 50, and 10 per cent (Dv(0.9), Dv(0.5), Dv(0.1)) of the used bulking agents; *n* = 3.

Bulking Agent	Dv(0.9) ± SD [µm]	Dv(0.5) ± SD [µm]	Dv(0.1) ± SD [µm]
m35	51.96 ± 0.84	13.41 ± 0.10	2.12 ± 0.05
la	116.94 ± 1.89	39.95 ± 0.37	6.13 ± 0.04
mcc	255.96 ± 3.84	113.70 ± 2.36	38.27 ± 0.46

**Table 4:** Bulk density ρ_b_ and compressed density ρ_c_ of the used bulking agents with the resulting mass of bulking agent m_b_ for 30 size #1 capsules (*) ($V_{c}$ = 0.5 mL) (size 1); *n* = 3.

Bulking Agent	$\rho_{b}\;\pm \;\mathrm{SD}\;[g/\mathrm{mL}]$	$\rho_{c}\;\pm \;\mathrm{SD}\;[g/\mathrm{mL}]$	Density Increase ± SD [%]	$m_{b}\;\pm \;\mathrm{SD}\;[g]\;*$
m35	0.531 ± 0.003	0.705 ± 0.011	24.7 ± 1.78	8.618 ± 0.072
la	0.564 ± 0.006	0.781 ± 0.027	27.7 ± 3.15	9.274 ± 0.170
mcc	0.392 ± 0.003	0.443 ± 0.002	11.3 ± 1.64	6.071 ± 0.038

**Table 5:** Quality parameters of baclofen 1 mg capsule batches prepared with bulking agents comprising m35, la and mcc; *n* = 30.

Batch	AV	Powder Loss [%]	SD_rel_ Mass Deviation [%]	SD_rel_ Content [%]	Mean Capsule Content [mg]	SD_rel_ Content/Mass [%]
m35-1mg-1	5.65	1.98	2.02	2.33	0.97	1.20
m35-1mg-2	4.78	2.40	1.88	2.37	1.01	1.41
m35-1mg-3	4.88	2.03	1.78	2.47	0.98	1.51
la-1mg-1	5.03	2.37	1.83	2.48	0.98	1.72
la-1mg-2	4.18	1.63	1.50	2.10	1.00	1.31
la-1mg-3	6.70	2.22	1.19	2.04	0.96	1.31
mcc-1mg-1	4.02	3.75	1.35	1.98	1.01	1.66
mcc-1mg-2	4.32	3.89	0.79	2.18	0.99	2.16
mcc-1mg-3	6.56	7.27	1.43	2.17	0.96	2.24

**Table 6:** Quality parameters of baclofen 2.5 mg capsule batches prepared with bulking agents comprising m35, la and mcc; *n* = 30.

Batch	AV	Powder Loss [%]	SD_rel_ Mass Deviation [%]	SD_rel_ Content [%]	Mean Capsule Content [mg]	SD_rel_ Content/Mass [%]
m35-2.5mg-1	3.08	4.22	1.64	1.56	2.46	0.552
m35-2.5mg-2	6.01	3.10	3.07	2.56	2.56	0.791
m35-2.5mg-3	4.92	2.53	3.02	2.46	2.51	0.962
la-2.5mg-1	5.15	1.95	2.13	2.57	2.50	1.15
la-2.5mg-2	3.86	2.17	1.54	1.50	2.56	1.62
la-2.5mg-3	2.66	1.69	1.41	1.30	2.54	0.947
mcc-2.5mg-1	16.07	1.31	1.43	6.36	2.61	6.44
mcc-2.5mg-2	5.54	4.67	1.36	2.50	2.55	2.13
mcc-2.5mg-3	7.42	1.93	1.24	2.03	2.62	1.78

**Table 7:** Quality parameters of baclofen 4 mg capsule batches prepared with bulking agents comprising m35, la and mcc; *n* = 30.

Batch	AV	Powder Loss [%]	SD_rel_ Mass Deviation [%]	SD_rel_ Content [%]	Mean Capsule Content [mg]	SD_rel_ Content/Mass [%]
m35-4mg-1	6.04	6.01	2.93	1.98	3.85	1.80
m35-4mg-2	3.50	4.72	0.79	1.63	3.93	0.891
m35-4mg-3	5.23	4.77	1.87	1.85	3.87	1.59
la-4mg-1	13.16	3.16	1.81	4.26	3.73	3.30
la-4mg-2	3.19	3.61	1.66	1.52	4.06	0.925
la-4mg-3	4.22	7.07	1.69	1.81	3.91	1.41
mcc-4mg-1	4.39	5.48	1.96	2.15	4.06	1.04
mcc-4mg-2	4.12	5.72	2.05	1.58	4.10	0.675
mcc-4mg-3	3.61	5.80	1.64	1.41	4.09	0.841

**Table 8:** Quality parameters of spironolactone 0.5 mg capsule batches prepared with bulking agents comprising m35, la and mcc; *n* = 30.

Batch	AV	Powder Loss [%]	SD_rel_ Mass Deviation [%]	SD_rel_ Content [%]	Mean Capsule Content [mg]	SD_rel_ Content/Mass [%]
m35-0.5mg-1	8.83	6.72	1.40	2.17	0.469	1.40
m35-0.5mg-2	11.29	10.67	2.19	2.31	0.457	1.67
m35-0.5mg-3	17.00	10.84	1.98	2.28	0.427	1.38
la-0.5mg-1	5.81	3.87	2.38	2.91	0.485	1.71
la-0.5mg-2	3.35	2.77	1.82	1.69	0.492	1.02
la-0.5mg-3	4.37	3.26	2.12	2.25	0.483	1.03
mcc-0.5mg-1	7.51	4.54	2.17	2.30	0.455	1.88
mcc-0.5mg-2	10.40	2.50	1.27	3.12	0.448	3.05
mcc-0.5mg-3	5.87	4.14	1.85	2.99	0.475	2.32

**Table 9:** Quality parameters of spironolactone 2 mg capsule batches prepared with bulking agents comprising m35, la and mcc; *n* = 30.

Batch	AV	Powder Loss [%]	SD_rel_ Mass Deviation [%]	SD_rel_ Content [%]	Mean Capsule Content [mg]	SD_rel_ Content/Mass [%]
m35-2mg-1	8.44	3.16	2.47	2.62	2.09	0.748
m35-2mg-2	4.24	4.66	2.39	2.12	2.00	0.704
m35-2mg-3	4.73	4.25	1.89	1.83	1.95	1.53
la-2mg-1	7.95	1.42	2.16	1.63	2.12	2.07
la-2mg-2	3.87	2.41	1.72	1.51	2.05	1.05
la-2mg-3	8.48	2.21	1.69	1.84	2.10	0.717
mcc-2mg-1	5.36	2.69	1.40	2.62	2.03	0.760
mcc-2mg-2	4.31	2.60	1.11	1.63	2.05	1.24
mcc-2mg-3	4.26	3.29	1.75	1.37	2.06	1.37

**Table 10:** Quality parameters of spironolactone 4 mg capsule batches prepared with bulking agents comprising m35, la and mcc; *n* = 30.

Batch	AV	Powder Loss [%]	SD_rel_ Mass Deviation [%]	SD_rel_ Content [%]	Mean Capsule Content [mg]	SD_rel_ Content/Mass [%]
m35-4mg-1	4.54	4.46	1.89	2.27	4.06	1.98
m35-4mg-2	10.59	2.44	3.90	4.06	4.16	0.821
m35-4mg-3	6.18	3.03	2.83	2.26	4.13	0.611
la-4mg-1	13.61	2.20	1.65	3.33	4.34	1.75
la-4mg-2	5.94	2.29	1.74	2.74	4.08	1.71
la-4mg-3	7.57	1.55	2.30	2.56	4.16	1.07
mcc-4mg-1	7.87	3.14	0.92	2.83	4.15	2.61
mcc-4mg-2	2.67	3.74	0.74	1.33	4.05	1.23
mcc-4mg-3	4.54	4.46	1.89	2.27	4.06	1.77

## Data Availability

Data is available from the author on request.
